# Protocol for phenotyping and isolation of dendritic cell subsets from blood and lymphoid organs of non-human primates and humans by flow cytometry

**DOI:** 10.1016/j.xpro.2025.103897

**Published:** 2025-06-13

**Authors:** Juliette Pons, Margaux Gardet, Florian Meurisse, Francis Relouzat, Olivier Lambotte, Benoit Favier

**Affiliations:** 1 Université Paris-Saclay, Inserm, CEA, Immunologie des Maladies Virales, Auto-immunes, Hématologiques et Bactériennes (IMVA-HB/IDMIT/UMRS1184), Fontenay-aux-Roses, 92265 Kremlin Bicêtre, France; 2 Paris-Saclay University Hospital Group, Assistance Publique Hôpitaux de Paris, Department of Internal Medicine and Clinical Immunology, Bicêtre Hospital, le Kremlin-Bicêtre, France

**Keywords:** Cell Biology, Cell isolation, Single Cell, Cell-based Assays, Flow Cytometry, Immunology, Molecular Biology

## Abstract

Dendritic cells (DCs) encompass several subsets that are essential for shaping immune responses. Here, we present a protocol for *ex vivo* functional analysis and purification of DC subsets from blood and secondary lymphoid organs using flow cytometry. We describe the steps for isolating mononuclear cells from blood, lymph nodes, and spleen. We then detail the procedures for phenotypic characterization of DC subsets, intracellular staining to assess their cytokine production, and fluorescence-activated cell sorting (FACS) to isolate individual DC populations.

For complete details on the use and execution of this protocol, please refer to Gardet et al.^1^

## Highlights

• Steps for isolating a single-cell suspension from blood, lymph nodes, and spleen of NHP
• Guidance on identifying DC subsets via flow cytometry with optimized antibody panels
• Steps for *ex vivo* stimulation and analysis of cytokine production by DC subsets
• Protocol for high-purity sorting of DC subsets for downstream assays

## Abstract (editor highlights)

Publisher’s note: Undertaking any experimental protocol requires adherence to local institutional guidelines for laboratory safety and ethics.

## Graphical abstract

**Figure:**
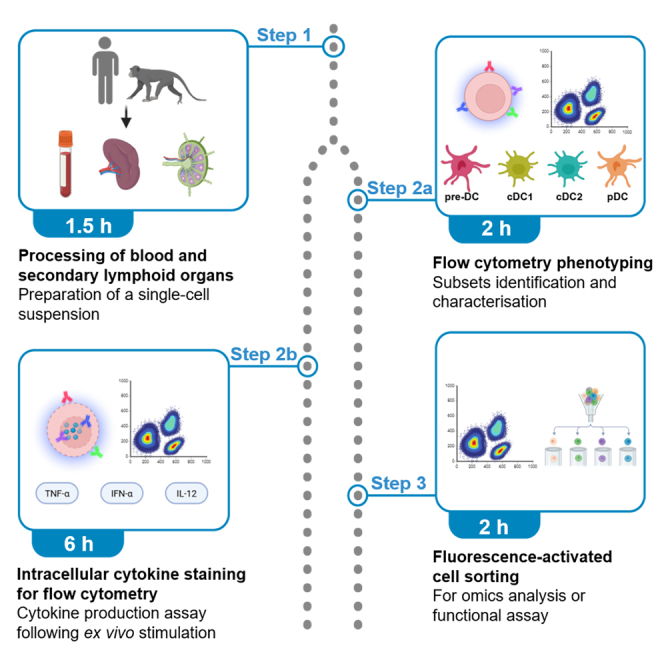

## Before you begin

Dendritic cells (DC) are composed of several subsets including pre-DC (also known as AS-DC), cDC1, cDC2, and pDC with mutual and exclusive functions that are essential to elicit proper immune responses.^2^^,^^3^^,^^4^ Dendritic cell subsets are found in various tissues where they serve as key sentinels of the immune system. After pathogen sensing, DC migrate toward secondary lymphoid organs through peripheral blood to shape immune responses. The protocol reported here describes the procedure for the direct analysis of non-human primate (NHP) dendritic cell (DCs) subsets from the peripheral blood, lymph nodes (LN) and spleen collected longitudinally (blood and LN) or at animal necropsies (blood, LN and spleen). This protocol is suitable to study DC subsets at homeostasis or to define changes in DC subsets numbers, phenotype and cytokine production capacity following viral infection of cynomolgus macaques. This protocol also includes an approach for high purity cell sorting of DC subsets from blood and LN that are suitable for transcriptomic analysis or *in vitro* assays in longitudinal studies.

For optimal results, secondary lymphoid organs samples should be stored immediately in cold processing medium (refer to materials and equipment section) and processed within 2 h of sampling. If not immediately processed after retrieval, secondary lymphoid organs should be stored whole at 4°C. Spleen and LN should be dissociated mechanically only, as enzymatic digestion is unnecessary. This protocol outlines two methods of mechanical dissociation: a manual method (see Steps 20–30), where organs are mashed over a filter, and an automated method using the GentleMACS dissociator (see Steps 12–19). Both approaches are suitable for spleen and LN; however, the manual method is more accessible, though it allows processing only one sample at a time. If working with more than three samples, we recommend using an automated tissue dissociator for efficiency.

Following puncture, peripheral blood should be stored in a Vacutainer tube with anticoagulant at room temperature and processed within 2 h of sampling. The use of heparin-lithium, EDTA, or citrate as anticoagulants is equally suitable for this process. The use of a CPT-tube to collect the blood is also an option.

Every procedure should be carried-out in a level 3 biosafety laboratory under a microbiological safety cabinet when the samples are coming from infected animals. Ensure the sterility of all materials used in this approach.

Here, we focus on the identification of pre-DC, cDC1, cDC2 and pDC subsets by flow cytometry in blood and secondary lymphoid organs of non-human primates. We provide a comprehensive list of titrated antibody clones used to build an optimized panel that discriminates between DC subsets. This panel also enables the assessment of their phenotype and their capacity to produce cytokines under *ex vivo* stimulation. We also provide a detailed protocol for the purification of DC subsets from peripheral blood and secondary lymphoid organs.

### Isolation of peripheral blood mononuclear cells

**Timing: 1 h**

Peripheral blood mononuclear cells (PBMCs) are isolated from whole blood using a density gradient medium. Remaining erythrocytes in the PBMCs suspension are then lysed and cells are counted before proceeding to further experiments. Volumes are indicated for an 8 mL blood sample in heparin-lithium anticoagulant.

***Note:*** Ensure all reagents and equipment are at 20°C.

1. Dilute the blood sample with PBS 1X to a 1:1 volume ratio.
2. Add 20 mL of 95% diluted Ficoll-Paque solution in a 50 mL Falcon.

**CRITICAL:** A density gradient medium with a density of 1.077 g/mL, such as Lymphoprep or Ficoll-Paque, is recommended. For Cynomolgus macaque blood, we dilute the density gradient medium by 5% with PBS 1X (for every 19 mL of Ficoll-Paque solution, add 1 mL of PBS 1X).

3. Carefully layer the diluted blood over the Ficoll-Paque without mixing the phases.
  a. Aspirate all the diluted blood with a pipette.
  b. Tilt the Falcon containing the Ficoll-Paque to an almost horizontal position.
  c. Stick the tip of the pipette as high as possible against the lower-wall of the Falcon.
  d. Release the blood from the pipette in a slow and steady stream.
  e. Once the blood reaches the Ficoll-Paque layer, slowly bring back the Falcon to a vertical position.
4. Centrifuge at 1,000 g for 20 min without acceleration or brake at 20°C.
5. Harvest the PBMC layer at the interface between the plasma and the Ficoll-Paque.
  a. Discard the upper layer of plasma using a pipette.
  b. Collect the cells in a new 50 mL Falcon using a pipette.
6. Top-up with PBS 1X and centrifuge at 200 g for 10 min at 20°C.
7. Aspirate supernatant and resuspend the pellet without adding any volume.
8. If required, add 5 mL of 37°C preheated RBC lysis solution 1X and incubate for 7 min at 20°C to lyse remaining red blood cells.
9. Top-up with PBS 1X and centrifuge at 200 g for 10 min at 20°C.
10. Aspirate supernatant and resuspend cell pellet in 1 mL of PBS 1X.

***Note:*** At that point, the cell pellet can be resuspended in the medium of choice for the follow-up experiment. To ensure optimal viability of the DC, try to keep as much as possible the sample at 4°C during all the rest of the experiment.

11. Evaluate cell concentration and viability using a manual or automated hemocytometer.

### Extraction of spleen mononuclear cells

**Timing: 1 h 20 min**

Spleen mononuclear cells (SMCs) are obtained through density separation of mechanically dissociated splenocytes to prepare a single-cell suspension.

12. Transfer the contents of the collection tube in a petri dish.
13. Using a scalpel and tweezers, cut the spleen in fragments of 1 cm^3^.
14. Split the fragments in 4 GentleMACS C tubes and add 2 mL of processing medium.
15. Load the tubes into the GentleMACS dissociator and execute 3 times a program of 60 s of dilaceration at 1260 rounds per run (rpr) at 20°C.
16. Place a 40 μm nylon filter over a 50 mL Falcon and humidify it with 1 mL of processing medium.
17. Transfer the contents of the GentleMACS tubes over the filter.
18. Add up to 30 mL of processing medium through the filter.
19. Proceed to density separation of SMCs by following the PBMC isolation protocol described in Steps 2 to 11. Split the sample across four 50 mL Falcon tubes for processing.

***Note:*** We observed that following density separation of splenocytes, the SMC layer appears to be slightly lower than the interface between the density gradient medium and the cell suspension.

### Separation of lymph node cells

**Timing: 10–20 min**

This section explains the manual mechanical dissociation of lymph nodes to prepare a single-cell suspension of lymph node cells (LNCs).

20. Place a 40 μm nylon filter over a 50 mL Falcon and humidify it with 1 mL of processing medium.
21. Transfer the contents of the collection tube in a petri dish.
22. Using a scalpel and tweezers, carefully remove the fat around the lymph nodes.
23. Split open the capsule of the lymph node using the scalpel and place it open-side down on the filter.
24. Using the plunger of a 10 mL syringe, gently mash the lymph node until the capsule is emptied.
25. Add processing medium regularly and stir until the capsule is emptied.
26. Rinse well both the plunger and the petri dish and top-up with processing medium through the filter.
27. Centrifuge at 200 g for 10 min at 4°C.
28. Carefully remove the supernatant by aspiration.
29. Resuspend the cell pellet in 1 mL of processing medium.
30. Transfer in a new 50 mL Falcon over a new humidified 40 μm filter and rinse with 4 mL of processing medium.

***Note:*** This second filtration step helps to remove cell clumps as well as fat deposits on the wall of the Falcon.

31. Evaluate cell concentration and viability using a manual or automated hemocytometer.

## Key resources table

**Table:** 

REAGENT or RESOURCE	SOURCE	IDENTIFIER
**Antibodies**		

Anti-human SynCAM (TSLC1/CADM1) (dilution 1/100)	CliniScience	Cat# CM004-3; RRID:AB_592783
Anti-human BDCA-2 APC (clone AC144) (dilution 1/50)	Miltenyi	Cat# 130113190 ; RRID:AB_2726015
Anti-human CD11b BV711 (clone ICRF44) (dilution 1/50)	BioLegend	Cat# 301344; RRID:AB_2563792
Anti-human CD123 PerCP-cy5.5 (clone 7G3) (dilution 1/50)	BD	Cat# 558714; RRID:AB_1645547
Anti-human CD14 V450 (clone M5E2) (dilution 3/100)	BD	Cat# 558121; RRID:AB_397041
Anti-human CD19 V450 (clone HIB19) (dilution 3/100)	Sony	Cat# 2111160
Anti-human CD1c APCcy7 (clone L161) (dilution 1/25)	BioLegend	Cat# 331520; RRID:AB_10644008
Anti-human CD20 V450 (clone L27) (dilution 3/100)	BD	Cat# 655872; RRID:AB_2870389
Anti-human CD3 V450 (clone SP34-2) (dilution 3/100)	BD	Cat# 560351; RRID:AB_1645168
Anti-human CD34 Pacific blue (clone 581) (dilution 1/50)	Sony	Cat# 2317560
Anti-human CD40 AF700 (clone 5C3) (dilution 1/25)	BioLegend	Cat# 334328; RRID:AB_2563920
Anti-human CD45 V500 (clone HI30) (dilution 1/50)	BD	Cat# 560777; RRID:AB_1937324
Anti-NHP CD45 V500 (clone D058-1283) (dilution 1/50)	BD	Cat# 561489; RRID:AB_10683313
Anti-human CD56 V450 (clone B159) (dilution 1/50)	BD	Cat# 560360; RRID:AB_1645578
Anti-human CD8 V450 (clone RPA-T8) (dilution 1/50)	BD	Cat# 560347; RRID:AB_1645581
Anti-human CD83 PE (clone HB15) (dilution 3/100)	BioLegend	Cat# 305322; RRID:AB_314516
Anti-human CD86 BUV737 (clone 2331) (dilution 3/100)	BD	Cat# 563412; RRID:AB_2916294
Donkey anti-chicken FITC (dilution 1/50)	Jackson ImmunoResearch	Cat# 703096155; RRID:AB_2340357
Anti-human HLA-DR BV605 (clone L243-G46-6) (dilution 1/200)	BioLegend	Cat# 307640; RRID:AB_2561913
Anti-human IFN-α PE (clone LT27:295) (dilution 1/25)	Miltenyi	Cat# 130-092-601; RRID:AB_871560
Anti-human IL-12/IL-23 p40 BIOTIN (clone C8.6) (dilution 3/100)	BioLegend	Cat# 508801; RRID:AB_315529
Anti-human Siglec-1 PE-VIO770 (clone 7–239) (dilution 1/50)	Miltenyi	Cat# 130-098-640; RRID:AB_2655549
Anti-human TNF-α AF700 (clone MAB11) (dilution 1/20)	BioLegend	Cat# 502928; RRID:AB_2561314
Streptavidin BV650 (1/100)	BioLegend	Cat# 405232

**Biological samples**		

Whole blood from cynomolgus macaques	IDMIT Facility	N/A
Tissues from cynomolgus macaques	IDMIT Facility	N/A

**Critical commercial assays**		

Foxp3/transcription factor staining buffer set	Invitrogen eBioscience	Cat# 00-5523-00
Perm/Wash 1X solution	Invitrogen eBioscience	Cat# 00-8333-56
Permeabilization buffer (10X)	Invitrogen eBioscience	Cat# 12766048
LIVE/DEAD fixable blue dead	Fisher Scientific	Cat# 10114812
EasySep PE positive selection kit II	STEMCELL	Cat# 17684
EasyEights EasySep magnet	STEMCELL	Cat# 18103
gentleMACS Octo dissociator	Miltenyi	Cat# 130-134-029
gentleMACS C tubes	Miltenyi	Cat# 130-093-237

**Software and algorithms**		

FACSDiva	BD Biosciences	https://www.bdbiosciences.com
FlowJo software v.10.0.8	BD Biosciences	https://www.flowjo.com
GraphPad Prism 8	GraphPad Software, Inc.	https://www.graphpad.com/scientific-software/prism/

**Other**		

Ficoll	Eurobio	Cat# CMSMSL01-01
Fetal bovine serum	Sigma-Aldrich	Cat# F7524
RPMI 1640 GlutaMAX	Gibco	Cat# 61870-010
Penicillin streptomycin neomycin	Gibco	Cat# 15640-055
Sodium pyruvate 1X	Thermo Fisher Scientific	Cat# 11360039
PBS	Gibco	Cat# 14190-094
R848/Resiquimod	Sigma	Cat# SML0196
BFA	Sigma	Cat# B-7651
NH4Cl	Sigma	Cat# 254134
KHCO_3_	Sigma	Cat# 60339
EDTA tetrasodium salt	Sigma	Cat# E5391
4% PFA fixation buffer	BioLegend	Cat# 420801

## Materials and equipment

**Table:** Processing medium

Reagent	Final concentration	Quantity for 1 L
RPMI 1640 GlutaMAX	N/A	900 mL
FBS	10% (v/v)	100 mL
EDTA tetrasodium salt	1 mM	0.38 g

Prepare freshly on the day of the experiment. Strain over a 0.2 μm filter.

**Table:** Staining buffer

Reagent	Final concentration	Quantity for 1 L
PBS	N/A	980 mL
FBS	2% (v/v)	20 mL
EDTA tetrasodium salt	1 mM	0.38 g

Strain over a 0.2 μm filter and aliquot after preparation. Store at −20°C for up to 6 months.

Thaw at 4°C overnight or at 20°C for a few hours. Can be stored at 4°C for up to 2 weeks.

**Table:** Culture medium

Reagent	Final concentration	Quantity for 1 L
RPMI 1640 GlutaMAX	N/A	880 mL
FBS	10% (v/v)	100 mL
Sodium pyruvate 100 mM	1 mM	10 mL
Penicillin, streptomycin & neomycin 100X	1X	10 mL

Prepare fresh on the day of the experiment. Strain over a 0.2 μm filter. Preheat at 37°C before use.

**CRITICAL:** FBS must be placed at 56°C for 30 min to inactivate immune complement and then strained through a 0.2 μm filter to remove protein aggregates. Store aliquots at −20°C for up to 6 months.

**Table:** Red blood cell lysis buffer 10X

Reagent	Quantity for 1 L
Distilled water	1 L
NH4Cl	82.6 g
KHCO_3_	10 g
EDTA tetrasodium salt	0.37 g

Weight each powder separately then add them to the distilled water, mix under agitation until dissolution. Adjust to a pH of 7.3. Aliquot in 50 mL Falcon and store at −20°C for up to 2 years.

### Red blood cell lysis buffer 1X

Thaw and dilute a 50 mL aliquot of 10X Red blood cell lysis buffer in 500 mL of distilled water. Strain using a 0.2 μm filter. Store at 4°C for up to 2 weeks. Preheat to 37°C before use.

## Step-by-step method details

### Phenotyping of DCs subsets

**Timing: 2 h**

This step outlines the flow cytometry staining for phenotyping DC subsets from PBMCs, SMCs, or LNCs. The entire procedure should be performed at 4°C using cold buffers, while avoiding direct light exposure. Make sure to prepare an unstained control tube (for Step 4, 7 and 10, cells are resuspended in staining buffer only). Volumes are indicated for a test on 5×10^6^ cells.

1. Transfer 5×10^6^ cells in a 5 mL FACS tube.
2. Top-up with staining buffer and centrifuge at 200 g for 5 min at 4°C, then remove supernatant by aspiration.
3. Prepare viability buffer by diluting 1 μL of LIVE/DEAD Fixable Blue Stain in 1 mL of PBS 1X.
4. Resuspend the pellet in 100 μL of viability buffer.
5. Incubate 15 min at 4°C.
6. Top-up with staining buffer and centrifuge at 200 g for 5 min at 4°C, then remove supernatant by aspiration.
7. Resuspend the pellet in 100 μL of antibody mix 1 in staining buffer (Table 1).

**Table 1:** Flow cytometry panel for DC subset phenotyping

Target	Clone	Host	Isotype	Conjugate	Laser	Filter	Dilution	Step
**Dead cells**	/	/	/	/	355 nm	U450/50	1/1000	**Viability buffer**
**Human CD32abc**	FLI8.26	mouse	IgG2b, κ	/	/	/	1/100	**Antibody mix 1**
**Human CADM1**	3E1	chicken	IgY	/	/	/	1/100	**Antibody mix 1**
**Human CD86**	2331	mouse	IgG1, κ	BUV737	355 nm	U740/35	3/100	**Antibody mix 2**
**Human CD3**	SP34-2	mouse	IgG1, λ	V450	405 nm	V450/50	3/100	**Antibody mix 2**
**Human CD8a**	RPA-T8	mouse	IgG1, κ	V450	405 nm	V450/50	1/50	**Antibody mix 2**
**Human CD14**	M5E2	mouse	IgG2a, κ	V450	405 nm	V450/50	3/100	**Antibody mix 2**
**Human CD20**	L27	mouse	IgG1, κ	V450	405 nm	V450/50	3/100	**Antibody mix 2**
**Human CD34**	581	mouse	IgG1, κ	Pacific blue	405 nm	V450/50	1/50	**Antibody mix 2**
**NHP CD45**	D058-1283	mouse	IgG1, κ	V500	405 nm	V525/50	1/50	**Antibody mix 2**
**Human HLA-DR**	L243-G46-6	mouse	IgG2a, κ	BV605	405 nm	V610/20	1/200	**Antibody mix 2**
**Human CD11b**	ICRF44	mouse	IgG1, κ	BV711	405 nm	V710/50	1/50	**Antibody mix 2**
**Chicken IgY**	polyclonal	donkey	F(ab’)_2_	FITC	488 nm	B530/30	1/50	**Antibody mix 2**
**Human CD123**	7G3	mouse	IgG2a, κ	PerCP-Cyanine5.5	488 nm	B695/40	1/50	**Antibody mix 2**
**Human CD83**	HB15e	mouse	IgG1, κ	PE	561 nm	Y585/15	3/100	**Antibody mix 2**
**Human SIGLEC1**	7-239	mouse	IgG1, κ	PE-Vio770	561 nm	Y780/60	1/50	**Antibody mix 2**
**Human BDCA2**	AC144	mouse	IgG1, κ	APC	640 nm	R670/14	1/50	**Antibody mix 2**
**Human CD40**	5C3	mouse	IgG1, κ	AF700	640 nm	R730/45	1/25	**Antibody mix 2**
**Human CD1c**	L161	mouse	IgG1, κ	APC-Cyanine7	640 nm	R780/60	1/25	**Antibody mix 2**

The viability buffer should be prepared by diluting 1 μL of LIVE/DEAD Fixable Blue Stain in 1 mL of PBS 1X.

For each test using 5×10^6^ cells, 100 μL of antibody mix 1 should be prepared by adding the CD32 blocking antibody and the primary antibody against CADM1 (Chicken, IgY) in staining buffer.

Similarly, 100 μL of antibody mix 2 should be prepared per test by adding the remaining primary antibodies of the panel along with the secondary antibody (Donkey anti-chicken IgY) to reveal CADM1 expression, also in staining buffer.
8. Incubate 30 min at 4°C, gently tap the tube every 10 min to resuspend the cell suspension.
9. Top-up with staining buffer and centrifuge at 200 g for 5 min at 4°C, then remove supernatant by aspiration.
10. Resuspend the pellet in 100 μL of antibody mix 2 in staining buffer (Table 1).
11. Incubate for 30 min at 4°C, gently tap the tube every 10 min to resuspend the cell suspension.
12. Top-up with staining buffer and centrifuge at 200 g for 5 min at 4°C, then remove supernatant by aspiration.
13. Resuspend the pellet in 200 μL of 1% paraformaldehyde in PBS 1X and incubate at least 15 min at 4°C to fix cells.
14. Acquire using the BD Fortessa cytometer in the following 12 h.

### Intracellular staining of cytokine production

**Timing: 6 h**

This step details the *ex vivo* stimulation of PBMCs, SMCs, or LNCs with R848 (Resiquimod, a TLR7 and TLR8 agonist) to measure intracellular cytokine production of DC subsets by flow cytometry staining. Always include an unstimulated cell control and prepare an unstained cell control for the permeabilized conditions.

15. Transfer 5×10^6^ cells in a 5 mL FACS tube.

***Note:*** For *ex vivo* stimulations, we recommend to include experimental triplicates for each condition.

16. Top-up with pre-heated culture medium and centrifuge at 200 g for 5 min at 20°C, then remove supernatant by aspiration.
17. Resuspend in 180 μL of culture medium with or without 10 μM of R848.
18. Incubate at 37°C in an incubator with 5% CO_2_.
19. After 2 h of incubation, add 20 μL of culture medium with 100 μg/mL of brefeldin A and gently mix (for a final concentration of 10 μg/mL of brefeldin A in 200 μL).
20. Incubate at 37°C in an incubator with 5% CO_2_ for 2 h (or more depending on your experiment requirement).
21. Top-up with cold staining buffer and centrifuge at 200 g for 5 min at 4°C, then remove supernatant by aspiration and wash again with cold staining buffer to ensure the complete removal of the culture medium to avoid the FBS to interact with the viability staining.

**CRITICAL:** From this point onward, perform the remainder of the procedure at 4°C using cold buffers, avoiding direct light exposure.

22. Proceed to extracellular staining of cells as described for phenotyping from Step 3 to 12:
  a. Prepare viability buffer by diluting 1 μL of LIVE/DEAD Fixable Blue Stain in 1 mL of PBS 1X.
  b. Resuspend the pellet in 100 μL of viability buffer.
  c. Incubate 15 min at 4°C.
  d. Top-up with staining buffer and centrifuge at 200 g for 5 min at 4°C, then remove supernatant by aspiration.
  e. Resuspend the pellet in 100 μL of antibody mix 1 in staining buffer (Table 2).

**Table 2:** Flow cytometry panel for measurement of intracellular cytokine production by DC subsets

Target	Clone	Host	Isotype	Conjugate	Laser	Filter	Dilution	Step
**Dead cells**	/	/	/	/	355 nm	U450/50	1/1000	**Viability buffer**
**Human CD32abc**	FLI8.26	mouse	IgG2b, κ	/	/	/	1/100	**Antibody mix 1**
**Human CADM1**	3E1	chicken	IgY	/	/	/	1/100	**Antibody mix 1**
**Human CD3**	SP34-2	mouse	IgG1, λ	V450	405 nm	V450/50	3/100	**Antibody mix 3**
**Human CD8a**	RPA-T8	mouse	IgG1, κ	V450	405 nm	V450/50	1/50	**Antibody mix 3**
**Human CD14**	M5E2	mouse	IgG2a, κ	V450	405 nm	V450/50	3/100	**Antibody mix 3**
**Human CD20**	L27	mouse	IgG1, κ	V450	405 nm	V450/50	3/100	**Antibody mix 3**
**Human CD34**	581	mouse	IgG1, κ	Pacific blue	405 nm	V450/50	1/50	**Antibody mix 3**
**NHP CD45**	D058-1283	mouse	IgG1, κ	V500	405 nm	V525/50	1/50	**Antibody mix 3**
**Human HLA-DR**	L243-G46-6	mouse	IgG2a, κ	BV605	405 nm	V610/20	1/200	**Antibody mix 3**
**Human CD11b**	ICRF44	mouse	IgG1, κ	BV711	405 nm	V710/50	1/50	**Antibody mix 3**
**Chicken IgY**	polyclonal	donkey	F(ab’)_2_	FITC	488 nm	B530/30	1/50	**Antibody mix 3**
**Human CD123**	7G3	mouse	IgG2a, κ	PerCP-Cyanine5.5	488 nm	B695/40	1/50	**Antibody mix 3**
**Human SIGLEC1**	7–239	mouse	IgG1, κ	PE-Vio770	561 nm	Y780/60	1/50	**Antibody mix 3**
**Human BDCA2**	AC144	mouse	IgG1, κ	APC	640 nm	R670/14	1/50	**Antibody mix 3**
**Human CD1c**	L161	mouse	IgG1, κ	APC-Cyanine7	640 nm	R780/60	1/25	**Antibody mix 3**
**Human IL-12p40**	C8.6	mouse	IgG1, κ	Biotin	/	/	3/100	**Antibody mix 4**
**Human IFN-α**	LT27:295	mouse	IgG1, κ	PE	561 nm	Y585/15	1/25	**Antibody mix 4**
**Human TNF-α**	Mab11	mouse	IgG1, κ	AF700	640 nm	R730/45	1/20	**Antibody mix 4**
**Biotin**	/	/	/	BV650	405 nm	V660/20	1/100	**Streptavidin buffer**

The viability buffer should be prepared by diluting 1 μL of LIVE/DEAD Fixable Blue Stain in 1 mL of PBS 1X.

For each test using 5×10^6^ cells, 100 μL of antibody mix 1 should be prepared by adding the CD32 blocking antibody and the primary antibody against CADM1 (Chicken, IgY) in staining buffer.

Similarly, 100 μL of antibody mix 3 should be prepared per test by adding the remaining extracellular primary antibodies of the panel along with the secondary antibody (Donkey anti-chicken IgY) to reveal CADM1 expression, also in staining buffer.

After fixation and permeabilization of cells, 100 μL of antibody mix 4 is prepared per test by adding the antibody targeting cytokines to reconstituted FoxP3/Transcription Factor Staining Buffer Set PermWash solution. Finally, streptavidin is diluted in PermWash solution for the detection and amplification of IL-12p40 staining. Prepare 100 μL of streptavidin buffer per test.
  f. Incubate 30 min at 4°C, gently tap the tube every 10 min to resuspend the cell suspension.
  g. Top-up with staining buffer and centrifuge at 200 g for 5 min at 4°C, then remove supernatant by aspiration.
  h. Resuspend the pellet in 100 μL of antibody mix 3 in staining buffer (Table 2).
    ***Note:*** Antibody mix 3 is sufficient for phenotyping DC subsets to quantify their cytokine production with antibody mix 4. Antibody mix 2 contains activation markers that are not required for this assay. If you wish to include activation markers (CD83, CD86, CD40) in this panel, ensure that the fluorochromes are compatible.
  i. Incubate for 30 min at 4°C, gently tap the tube every 10 min to resuspend the cell suspension.
  j. Top-up with staining buffer and centrifuge at 200 g for 5 min at 4°C, then remove supernatant by aspiration.
23. Resuspend cells in 200 μL of Permeabilization Buffer and incubate for 20 min at 4°C.
24. Top-up with PermWash solution and centrifuge at 200 g for 5 min at 4°C, then remove supernatant by aspiration.
25. Resuspend the pellet in 100 μL of antibody mix 4 in PermWash solution (Table 2).
26. Incubate for 30 min at 4°C, gently tap the tube every 10 min to resuspend the cell suspension.
27. Top-up with PermWash solution and centrifuge at 200 g for 5 min at 4°C, then remove supernatant by aspiration.
28. Resuspend the pellet in 100 μL of streptavidin reagent in PermWash solution (Table 2).
29. Incubate for 30 min at 4°C.
30. Top-up with PermWash solution and centrifuge at 200 g for 5 min at 4°C, then remove supernatant by aspiration.
31. Resuspend in 200 μL of 1% paraformaldehyde in PBS 1X.
32. Acquire using the BD Fortessa cytometer in the following 12 h.

### Fluorescence-activated cell sorting of DC subsets

**Timing: 2 h**

This section details how DC subsets from PBMCs, SMCs or LNCs can be purified with a fluorescence-activated cell sorting approach. Cells are first enriched in DCs through magnetic depletion of unwanted populations, then fully stained and sorted using a BD AriaFusion cytometer. The sorted DC populations can be further used in functional assays, for example, their ability to induce T-cell proliferation can be measured in mixed leukocyte reaction (MLR) assays. Such purified DC populations can also be used for bulk or single-cell transcriptomic or proteomic analyses.

33. Prepare collection tubes to harvest sorted cells.
  a. Coat 1.5 mL Eppendorf tubes with FBS at 37°C for 2 h.
  b. Decant the FBS from the Eppendorf tubes just before sorting, leaving about 50 μL to retain a minimal volume for receiving the sorted cells.
34. Magnetic depletion of unwanted populations using StemCell EasySep PE Positive Selection kit.
  a. Transfer 100×10^6^ cells in FACS tube.
  b. Top-up with staining buffer and centrifuge at 200 g for 5 min at 4°C, then remove supernatant by aspiration.
  c. Resuspend pellet in 400 μL of antibody mix 1 in staining buffer (Table 3).

**Table 3:** Flow cytometry panel for live-cell sorting of DC subsets

Target	Clone	Host	Isotype	Conjugate	Laser	Filter	Dilution	Step
**Human CD32abc**	FLI8.26	mouse	IgG2b, κ	/	/	/	1/100	**Antibody mix 1**
**Human CADM1**	3E1	chicken	IgY	/	/	/	1/100	**Antibody mix 1**
**Human CD3**	SP34-2	mouse	IgG1, λ	PE	561 nm	Y585/15	3/100	**Antibody mix 5**
**Human CD8a**	RPA-T8	mouse	IgG1, κ	PE	561 nm	Y585/15	1/50	**Antibody mix 5**
**Human CD14**	M5E2	mouse	IgG2a, κ	PE	561 nm	Y585/15	3/100	**Antibody mix 5**
**Human CD20**	L27	mouse	IgG1, κ	PE	561 nm	Y585/15	3/100	**Antibody mix 5**
**Human CD34**	581	mouse	IgG1, κ	PE	561 nm	Y585/15	1/50	**Antibody mix 5**
**Dead cells**	/	/	/	/	355 nm	U450/50	1/1000	**Viability buffer**
**NHP CD45**	D058-1283	mouse	IgG1, κ	V500	405 nm	V525/50	1/50	**Antibody mix 6**
**Human HLA-DR**	L243-G46-6	mouse	IgG2a, κ	BV605	405 nm	V610/20	1/200	**Antibody mix 6**
**Human CD11b**	ICRF44	mouse	IgG1, κ	BV711	405 nm	V710/50	1/50	**Antibody mix 6**
**Chicken IgY**	polyclonal	donkey	F(ab’)_2_	FITC	488 nm	B530/30	1/50	**Antibody mix 6**
**Human CD123**	7G3	mouse	IgG2a, κ	PerCP-Cyanine5.5	488 nm	B695/40	1/50	**Antibody mix 6**
**Human SIGLEC1**	7–239	mouse	IgG1, κ	PE-Vio770	561 nm	Y780/60	1/50	**Antibody mix 6**
**Human BDCA2**	AC144	mouse	IgG1, κ	APC	640 nm	R670/14	1/50	**Antibody mix 6**
**Human CD1c**	L161	mouse	IgG1, κ	APC-Cyanine7	640 nm	R780/60	1/25	**Antibody mix 6**

The viability buffer should be prepared by diluting 1 μL of LIVE/DEAD Fixable Blue Stain in 1 mL of PBS 1X.

For each test using 100×10^6^ cells, 400 μL of antibody mix 1 should be prepared by adding the CD32 blocking antibody and the primary antibody against CADM1 (Chicken, IgY) in staining buffer.

Then, 100 μL of antibody mix 5 should be prepared per test by adding the primary antibodies targeting lineage markers also in staining buffer.

Finally, 250 μL of antibody mix 6 should be prepared per test by adding the remaining primary antibodies of the panel along with the secondary antibody (Donkey anti-chicken IgY) to reveal CADM1 expression, also in staining buffer.
  d. Incubate 30 min at 4°C.
  e. Without washing, add 100 μL of antibody mix 5 in staining buffer (Table 3).
  f. Incubate 30 min at 4°C.
  g. Top-up with staining buffer and centrifuge at 200 g for 5 min at 4°C, then remove supernatant by aspiration.
  h. Resuspend in 460 μL of staining buffer.
  i. Take aside 10 μL of cell suspension that will later be used for pre-enrichment control.
  j. Add 50 μL of Selection cocktail and incubate 15 min at 4°C.
    **CRITICAL:** Do not vortex the Selection cocktail stock solution, instead, gently pipette up and down to homogenize.
  k. Add 50 μL of RapidSpheres and incubate 10 min at 4°C.
  l. Add 2 mL of staining buffer and place in the magnet, top off, for 10 min.
  m. Carefully remove the volume with a 2 mL pipette without touching the part of the tube in contact with the magnet and transfer it into a new FACS tube.
  n. Top-up with staining buffer and centrifuge at 200 g for 5 min at 4°C, then remove supernatant by aspiration.
35. Prepare viability buffer by diluting 1 μL of LIVE/DEAD Fixable Blue Stain in 1 mL of PBS 1X.
36. Resuspend the pellet in 250 μL of viability buffer.
37. Incubate 15 min at 4°C.
38. Top-up with staining buffer and centrifuge at 200 g for 5 min at 4°C, then remove supernatant by aspiration.
39. Resuspend the pellet in 250 μL of antibody mix 6 in staining buffer (Table 3).
40. Incubate for 30 min at 4°C, gently tap the tube every 10 min to resuspend the cell suspension.
41. Top-up with processing medium and centrifuge at 200 × *g* for 5 min at 4°C, then remove supernatant by aspiration.
42. Resuspend the pellet in 500 μL of processing medium.

**Pause point:** At this point, when working with multiple samples at the same time, stained cells are stored at 4°C until sorting.

43. Right before sorting, gently resuspend and strain over a 50 μm BD Filcon cup-type filter.
44. Sort using a 100 μm nozzle at 20 psi, with 4-way purity precision mode, make sure flow rate stays under 6,000 events/second for the duration of the sort.

**CRITICAL:** The sorter used should be equipped with a cooling module to ensure that all fluidics and collection tubes are kept cold for the duration of the sort.

## Expected outcomes

**Figure 1:**
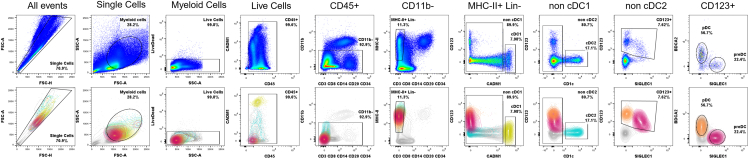
Complete gating strategy for identifying DC subsets in NHP peripheral blood Representative gating strategies to identify DC subsets among PBMCs are shown using pseudocolor density plots and 5% colored contour plots. Each dendritic cell subset can be followed in the gating strategy: pDC (orange); pre-DC (pink); cDC1 (yellow); cDC2 (blue).

The procedures described here enable the robust identification of four distinct DC subsets in peripheral blood and secondary lymphoid organs (Figure 1). This standardized flow cytometry panel and gating strategy can be applied across different tissues and species, ensuring reproducible and comparable data.

**Figure 2:**
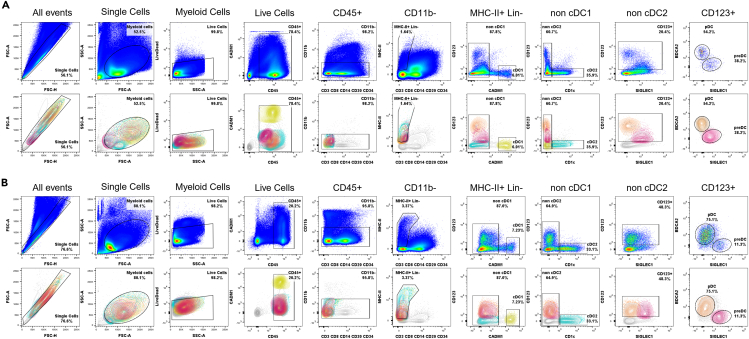
Complete gating strategy for identifying DC subsets in NHP secondary lymphoid organs Representative gating strategies to identify DC subsets among SMCs (A) and LNCs (B) are shown using pseudocolor density plots and 5% colored contour plots for each tissue type. Each dendritic cell subset can be followed in the gating strategy: pDC (orange); pre-DC (pink); cDC1 (yellow); cDC2 (blue).

Singlets are first identified by plotting FSC-H against FSC-A, followed by gating on FSC-A vs. SSC-A to distinguish myeloid cells by morphology. Live leukocytes are selected as CD45-positive and negative for LIVE/DEAD Fixable Blue Stain. Monocytes (in peripheral blood, Figure 1) and macrophages (in secondary lymphoid organs, Figure 2) are excluded based on CD11b expression. Unwanted populations are further removed using lineage markers: CD3 and CD8a for T cells, CD8a for NK cells, CD20 for B cells, and CD34 for hematopoietic stem cells. Any remaining CD11b-negative monocytes or macrophages are excluded based on CD14 expression.

Following these steps, DCs are identified as MHC-II-positive (HLA-DR^+^) events that do not express lineage markers. The cDC1 subset (yellow) is distinguished by its expression of CADM1, which is identified by plotting CD123 against CADM1.^5^ Within the CADM1-negative population, the cDC2 subset (blue) is identified based on CD1c expression. To distinguish pDCs (orange) and pre-DCs (pink), the remaining CADM1- and CD1c-negative DCs are first plotted for CD123 against SIGLEC1, allowing for the identification of CD123-positive populations. Finally, CD123-positive DCs are further analyzed by plotting BDCA2 against SIGLEC1, where pDCs are identified as CD123-high, SIGLEC1-negative, and BDCA2-positive, whereas pre-DCs are characterized as CD123-low, SIGLEC1-positive, and BDCA2-negative.^1^^,^^6^

Further information on expected frequencies and phenotypic marker expression, such as CD86, CD83 and CD40, can be found in previously published research articles that used this protocol.^1^^,^^7^ These studies also report cytokine production by DC subsets, including IL-12p40, IFN-α and TNF-α.

## Limitations

Although the gating strategy presented here for NHP cells is also suitable for human cells, some modifications to the antibody panel should be considered when staining human samples. Differences in cross-species antibody reactivity can affect certain targets, as seen with CD45. Specifically, human leukocytes should be identified using anti-human CD45 (clone HI30), as its reactivity differs from the NHP counterpart.

Additionally, lineage marker expression varies between NHPs and humans. For example, CD56 is a more suitable marker for excluding NK cells in human samples compared to CD8, which is commonly used in NHPs. Similarly, B-lymphocyte identification differs between species, as CD19 is broadly expressed in human B cells, whereas CD20 provides broader coverage in NHPs.

When working with samples from both species, we recommend including both CD45 clones conjugated to the same fluorophore to ensure consistent leukocyte identification. Furthermore, to refine lineage exclusion, human-specific CD56 (clone B159) and CD19 (clone HIB19) should be added to the panel. These adjustments help optimize the protocol for comparative studies involving both NHP and human samples.

We were unable to identify the DC3 subset in NHP. In humans, DC3s express CD14, so CD88 is typically used to exclude monocytes from this population.^8^ However, there is currently no cross-reactive anti-CD88 antibody that works reliably in NHP. Although BTLA and CD5 have also been proposed as markers to help identify DC3s, their staining patterns in NHP were inconclusive.

## Troubleshooting

### Problem 1

Red blood cell contamination in the mononuclear cell layer at the interphase after density gradient centrifugation. Step 5 of isolation of peripheral blood mononuclear cells.

### Potential solution

Red blood cell contamination in the mononuclear cell layer at the interphase often results from insufficient density gradient separation. To prevent this, ensure the density gradient medium is at 20°C before use and carefully layer the blood sample to avoid mixing. Improper centrifugation can also disrupt the gradient, so using a soft brake or no brake is essential to maintain layer integrity. Additionally, overloading the gradient medium should be avoided; blood volume should not exceed 50% of the total gradient volume, and large samples should be split into multiple tubes.

Non-human primate red blood cells differ in density and behavior from human red blood cells, affecting separation efficiency. To mitigate contamination, we dilute the density gradient medium with 1 mL of PBS per 19 mL of medium, slightly lowering its density and improving mononuclear cell recovery.

### Problem 2

Cloudy and sticky single-cell suspension following isolation from peripheral blood or spleen. Step 11 of isolation of peripheral blood mononuclear cells.

### Potential solution

Platelets are inherently sticky and tend to form clumps with other cell types, leading to aggregation in mononuclear cell suspensions. To minimize platelet contamination after density gradient centrifugation, carefully remove the upper, platelet-rich plasma layer without disturbing the mononuclear cell layer at the interphase. Additionally, perform one or more slow spin washes (120 g, 10 min, brake off, 20°C) until excess platelets are effectively removed, ensuring a cleaner, more homogenous suspension.

### Problem 3

Insufficient cell pelleting after centrifugation. Step 6 of isolation of peripheral blood mononuclear cells.

### Potential solution

Low-speed centrifugation effectively removes cell debris and dead cells without damaging the cell pellet. We found that centrifuging for 5 min at 200 g in a swinging-bucket centrifuge (Thermo Scientific Rotor Swing-Out TX-1000) is sufficient to pellet cells in 5 mL FACS tubes. For 15 mL and 50 mL Falcon tubes, increase the centrifugation time to 10 min to ensure complete pelleting.

To minimize pellet disturbance, we recommend reducing the brake setting to 7 out of 9, which helps mitigate acceleration forces and angle changes.

If using a fixed-angle rotor centrifuge, consider increasing the centrifugation speed to compensate for differences in pelleting efficiency.

When working with a low number of cells (such as during the staining procedure), use a V-bottom 96-well plate to ensure efficient pellet formation.

Additionally, always ensure that buckets are properly balanced to reduce vibrations and improve consistency, even small imbalances may dislodge the pellet.

### Problem 4

Difficulty completing cell suspension preparation, stimulation and staining in a single day due to time constraints. Step 26 of intracellular staining of cytokine production.

### Potential solution

When working with fresh samples, especially for ex vivo stimulation of DC subsets, it can be challenging to complete the entire workflow the same day. To address this, the intracellular staining step can be performed overnight, as demonstrated previously.^9^ After stimulation and extracellular staining, cells can be fixed and permeabilized before proceeding with intracellular staining overnight, helping to ease time pressure without compromising data quality.

### Problem 5

Clogged or unstable stream during live-cell sorting. Step 44 of fluorescence-activated cell sorting of DC subsets.

### Potential solution

To prevent clogging of the flow cell, filter all samples immediately before sorting using a 50 μm BD Filcon cup-type filter or an equivalent mesh filter. Additionally, to minimize cell clumping, incorporate EDTA (up to 2 mM) in the staining and processing medium. This helps prevent calcium-mediated cell-cell adhesion, ensuring a stable and efficient sorting process.

## Resource availability

### Lead contact

Further information and requests for resources and reagents should be directed to and will be fulfilled by the lead contact, Benoit FAVIER, (benoit.favier@cea.fr).

### Technical contact

Questions about the technical specifics of performing the protocol should be directed to the technical contact, Margaux GARDET, (margauxgardet@gmail.com).

### Materials availability

This study did not generate new unique reagents.

### Data and code availability

This study did not generate datasets or code.

## Acknowledgments

This work was supported by the Programme Investissements d’Avenir (PIA), managed by the ANR under reference ANR-11-INBS-0008, funding the Infectious Disease Models and Innovative Therapies (IDMIT, Fontenay-aux-Roses, France) infrastructure, and ANR-10-EQPX-02-01, funding the FlowCyTech facility (IDMIT, Fontenay-aux-Roses, France); the French National Agency for AIDS, Hepatitis and Emerging Infectious Disease (ANRS-MIE; grant number: ECTZ159192); and Sidaction (grant number: 21-1-AEQ-12963). We thank the staff of the animal facility of IDMIT, particularly B. Delache, S. Langlois, J.M. Robert, Q. Sconosciuti, and N. Dhooge. We also thank W. Gros, M. Leonec, M. Van Tilbeurgh, and A.S. Gallouet for flow cytometry assistance. The graphical abstract was created in BioRender (https://BioRender.com).

## Author contributions

Methodology, J.P., M.G., F.M., and B.F.; investigation, M.G.; resources, F.R.; writing – original draft, J.P.; writing – review and editing, J.P., M.G., F.M., and B.F.; supervision, O.L. and B.F.; funding acquisition, O.L. and B.F.

## Declaration of interests

The authors declare no competing interests.
